# Description of events reported to the Australian National Focal Point, 2014–2023

**DOI:** 10.5365/wpsar.2026.17.1.1335

**Published:** 2026-03-25

**Authors:** Amish Talwar, Martyn D Kirk

**Affiliations:** a National Centre for Epidemiology and Population Health, The Australian National University, Canberra, Australian Capital Territory, Australia.

The 2005 revision of the International Health Regulations (IHR; 2005) requires States Parties of the World Health Organization (WHO) to designate National Focal Points (NFPs) to serve as defined points of contact to communicate with WHO (Article 6: IHR notification) and other States Parties (Article 44: Collaboration and assistance) on IHR matters, particularly the mandatory reporting of significant public health events, including public health emergencies of international concern (PHEICs). (*1*) Although previous studies have evaluated the timeliness of event reporting from the start of an event to when the event information was disseminated through WHO’s *Disease Outbreak News*, (*2*, *3*) it remains less clear what types of events are reported to the NFPs and how those events are adjudicated, including whether to notify WHO of the event. WHO has recently promoted the 7–1-7 targets for detecting infectious disease threats within 7 days, notifying public health authorities within 1 day and initiating response activities within 7 days after being notified. (*4*, *5*) Ideally, to align with these targets, event information should include the times of event start, NFP notification and response initiation.

In Australia, the NFP function is hosted under the auspices of the Australian Government Department of Health, Disability and Ageing’s National Incident Centre (NIC), which supports national coordination of health sector emergencies. For diseases on the National Notifiable Disease List (NNDL), (*6*) the primary role of the Australian NFP for most domestic events is to provide notifications to Australian states and territories (or Responsible Bodies, which have constitutional authority for health responses), who may take appropriate public health action as per their legislative obligations or guidelines. Additionally, the NFP manages IHR (2005)-related communications between Australia and WHO as well as other governments. Following the COVID-19 pandemic and subsequent amendments to the IHR (2005), including a new requirement for a National IHR Authority to coordinate IHR (2005) implementation, (*7*) it is an opportune time for Australia to review events reported to its NFP and to describe its event information management system. In this Brief Report, we summarize events reported to the Australian NFP from 2014 to 2023.

## Methods

In Australia, information about events reported to the NFP is uploaded, stored and categorized in a dedicated incident management system (IMS). The main events are categorized as being reported from within Australia or by international NFPs, associated with flights or ships travelling domestically or internationally, or not otherwise classified. For our analysis, we used the following event information: date the NFP was notified; event category; type of associated hazard; registration of the event in the NNDL; name of the original notifying body; international NFPs notified; and domestic Responsible Bodies notified. Other event information, including date of event start, date of event detection, date of response initiation, place of event start and number of cases, was not directly available (or provided) through the IMS. Additionally, it was unclear which events were communicated to the WHO Regional Office for the Western Pacific. From the information made available, we determined the number of events by year and hazard type. Within these categories, we also reported the number of events according to whether they were reported from within or outside Australia. Among the events reported from within Australia, we determined whether these events were reported to other country NFPs.

## Results

From 2014 to 2023, 967 events were reported to the Australian NFP. Excluding two consolidated entries for international reporting for the COVID-19 pandemic and the monkeypox virus (mpox) multicountry epidemic, six events of unknown origin and eight non-notifications, 951 events were reported. From within Australia, 340/951 (35.8%) events were reported. These included events reported by an Australian state or territory that were associated with domestic flights and international flights originating in Australia. From outside Australia, 611/951 (64.2%) events were reported, including those reported from overseas or associated with flights originating and departing overseas, as well as flights and ships arriving in Australia. The greatest number of events was reported in 2020 (171/951, 18.0%) and the least in 2021 (19/951, 2.0%). The top three hazards reported from within Australia included tuberculosis (112/340, 32.9%), legionellosis (69/340, 20.3%) and measles (53/340, 15.6%) (**Table 1**).

## Discussion

This report presents the first extended description of events received or reported by an NFP. Among all hazards reported, tuberculosis, legionellosis and measles were the most reported by a substantial margin, both from within and outside Australia. This high reporting is likely a product of overseas acquisition of these diseases among people who travelled to Australia (with disease detection either at the point of origin, at a place of transit or upon arrival) and also reflects the NFP’s role in assisting with contact tracing of exposed travellers, indicating heightened concern over transmission of these particular diseases. (*8*-*10*) On the other hand, the decline in reporting from 2020 to 2021 is likely attributable to international travel and trade restrictions and the imposition of lockdowns during the COVID-19 pandemic. (*11*)

Although the Australian IMS provides an accurate ledger of events based on the type of event, when the Australian NFP was notified and whether other NFPs were notified, it does not consistently provide crucial information to evaluate the event reporting system in Australia, including the dates of event start, detection and response initiation. This makes it challenging to assess the efficacy of the reporting system in relation to the 7–1-7 standard and to compare it with other countries. Also, it is unclear from the IMS data why certain events were reported to NFPs in other countries while others were not. While the most recent version of the IHR (2005) Annex 2 lists a handful of diseases always requiring notification, NFPs must use the Annex 2 decision algorithm for all other potential PHEICs. (*1*) A 2011 study revealed that, when presented with fictitious public health events, NFPs did not necessarily agree with each other or an expert panel on which events required notification. (*12*) Finally, it is unclear from the available IMS data which events were communicated to the Regional Office and the rationale for doing so. These data would have been a clearer signal of events that the NFP adjudged as meeting the Annex 2 criteria for notification to WHO.

For a better understanding of why NFPs might have different reporting behaviours, NFPs should strive to document their rationale for reporting certain events and routinely compare their reporting behaviours to identify best practices and areas requiring remediation. The establishment of National IHR Authorities represents an opportune moment for NFPs to revisit how public health events are reported and tracked, and to update their data management systems to ensure accurate and comprehensive information gathering in line with State Parties’ IHR (2005) obligations. For a federal system like Australia, this would be an opportunity to examine how best to facilitate outbreak data centralization to comply with these practices amidst the devolution of public health responsibilities to states and territories, including the prerogative for what and when to report to the Australian NFP. Such an effort would represent a significant step forward for future pandemic prevention, preparedness and response.
